# A rare case of ciliated muconodular papillary tumor accompanied with adenocarcinoma in situ

**DOI:** 10.1186/s12890-021-01581-9

**Published:** 2021-07-12

**Authors:** Lei Zhao, Conner M. Willson, Nathan T. Givens, Ziwen Zhu, Mark R. Wakefield, Yongsheng Wang, Wanchun Yang, Yujiang Fang

**Affiliations:** 1 Department of Respiratory Medicine, The 2nd People’s Hospital of Hefei and Hefei Hospital Affiliated to Anhui Medical University, Hefei, China; 2 Department of Microbiology, Immunology and Pathology, Des Moines University College of Osteopathic Medicine, Des Moines, IA 50312 USA; 3 Department of Surgery, University of Missouri School of Medicine, Columbia, MO 65212 USA

**Keywords:** Ciliary muconodular papillary tumor, Adenocarcinoma in situ, Interstitial inflammation, Basal cells, Ciliated columnar epithelial cells, Type I End Respiratory Failure, Lung cancer

## Abstract

**Background:**

Ciliated muconodular papillary tumor (CMPT) is an incredibly rare pulmonary tumor. Currently, little is known about CMPT, and it has not yet been classified by the World Health Organization. The clinical manifestation of CMPT is nonspecific and the diagnosis is only based on pathology. CMPT has been documented in limited reports as a benign tumor, thus the treatment is typically with surgical excision if a solid tumor is identifiable. The prognosis of CMPT is very positive, as no recurrence has been reported in the limited literature available. However, CMPT accompanied with adenocarcinoma in situ has not been reported previously in the literature.

**Case presentation:**

In this report, we presented a case of a 53-year-old male smoker with CMPT associated with adenocarcinoma in situ. This diagnosis was confirmed by pathological examination, including immunohistostaining. No solid resectable lesion was identified on CT scan; therefore, no surgery was performed. The patient’s adenocarcinoma in situ was disseminated in both lungs, thus chemotherapeutic treatment with cisplatin and pemetrexed was given. The patient will be continually followed up closely on a wait-and-watch basis.

**Conclusions:**

In summary, our report reveals a unique case of CMPT in conjunction with adenocarcinoma in situ, potentially revealing an association between CMPT and malignancy which has not been previously reported. More similar case studies will be beneficial to determine the authentic relationship between CMPT and adenocarcinoma in situ.

**Supplementary Information:**

The online version contains supplementary material available at 10.1186/s12890-021-01581-9.

## Introduction

Ciliated muconodular papillary tumor (CMPT) is a very rare pulmonary tumor first reported by a Japanese physician, Dr. Ishikawa in 2002 [1]. In this initial case, the patient was a 50-year-old-female with a significant smoking history^1^. The tumor was located in the periphery of the right upper lung lobe and was 15 mm in size. Lobectomy was performed and at subsequent 10 year follow up with biopsy, there were no signs of recurrence of CMPT [2].

To date, there have only been 60 reported cases of CMPT [2, 3]. A prevalence of CMPT has been observed in East Asian populations [2, 4, 5]. Although most of the reported tumors have been seen in the periphery of the lung lobe, computed tomography (CT) has revealed a broad range of radiographic findings including small lung nodules, ground-glass opacity, or irregular-shaped consolidations [6]. In addition, there are a wide array of clinical presentations and sometimes indistinguishable pathological features of CMPT, which can ultimately lead to misdiagnosis of this rare tumor [3]. Due to the limited cases reported, the clinical diagnosis and management is still a challenge for clinicians. Thus, more information is needed to fully understand this rare disease.

Here, we report a unique case of CMPT accompanied with adenocarcinoma in situ found in a patient who had type I end respiratory failure and chronic fatty liver disease. There are few reports on this unique tumor, which has not yet received a WHO classification [2, 3, 6, 7]; this case adds to the body of knowledge about this disease and introduces a potential link to malignancy.

## Case presentation

Our patient is a 53-year-old male with a two-year history of hypertension and a 5-year history of hepatic steatosis and hyperlipidemia. He presented initially with a two-year history of cough with recent aggravation. The patient did not report any sputum production and denied any worsening of the cough with seasonal or environmental triggers. He reported associated chest tightness and wheezing with his cough. The patient did not have any history of surgery, trauma, or tuberculosis. He is a 15-pack year smoker. He works as a bricklayer. The patient was evaluated initially for pulmonary interstitial inflammation and subsequently underwent a two-week course of cephalosporin (dosage: 2.0 g, q8h). This treatment was not effective at improving his condition. During this time, he reported additional symptoms of anorexia, malaise, insomnia, and weight loss.

He was admitted to the hospital with the diagnosis of type 1 respiratory failure, following his previous course of treatment. The patient underwent more diagnostic tests to determine the source of his symptoms. His physical examination was largely unremarkable, with the exception of coarse breath sounds auscultated in both lungs. Arterial blood gas (ABG) evaluation upon admission showed hypocapnia (33.9 mmHg, reference range: 35–45 mmHg) and severe hypoxemia (40.3 mmHg, reference range 80–100 mmHg). The patient’s complete blood count (CBC) revealed leukocytosis (12.32 × 10^9^/L, reference range: 4.0–11.0 × 10^9^/L) and neutrophilia (9.93 × 10^9^/L, reference range: 2.0–8.0 × 10^9^/L), otherwise unremarkable. The patient’s basic metabolic panel (BMP) was all within normal ranges. No abnormalities on screenings for hepatitis, HIV, syphilis, or rheumatoid arthritis. He additionally had an elevated carcinoembryonic antigen (21.77 ng/mL, reference range: 0–5 ng/mL).

**Fig. 1:**
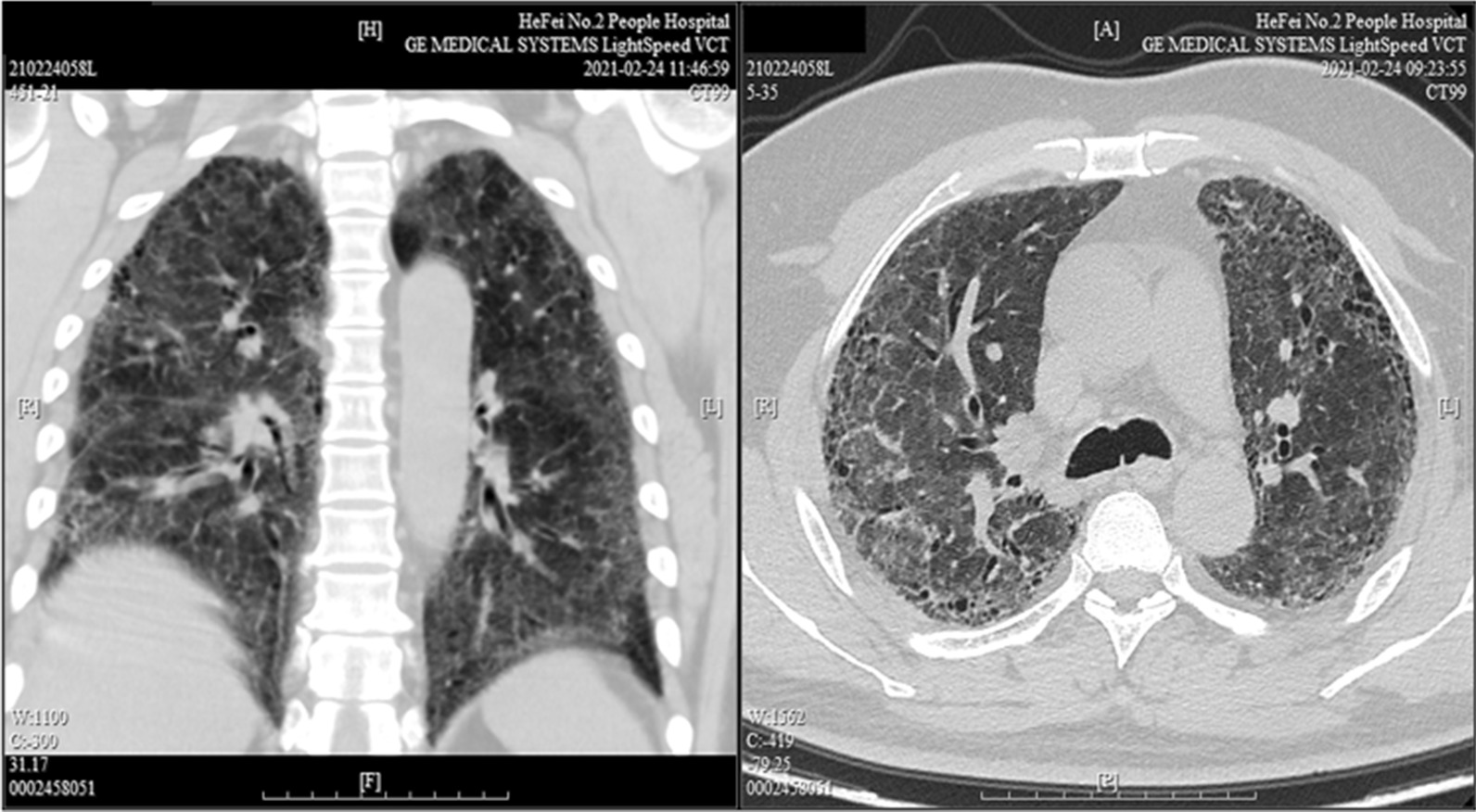
Chest CT suggesting bilateral inflammation in the lungs

**Fig. 2:**
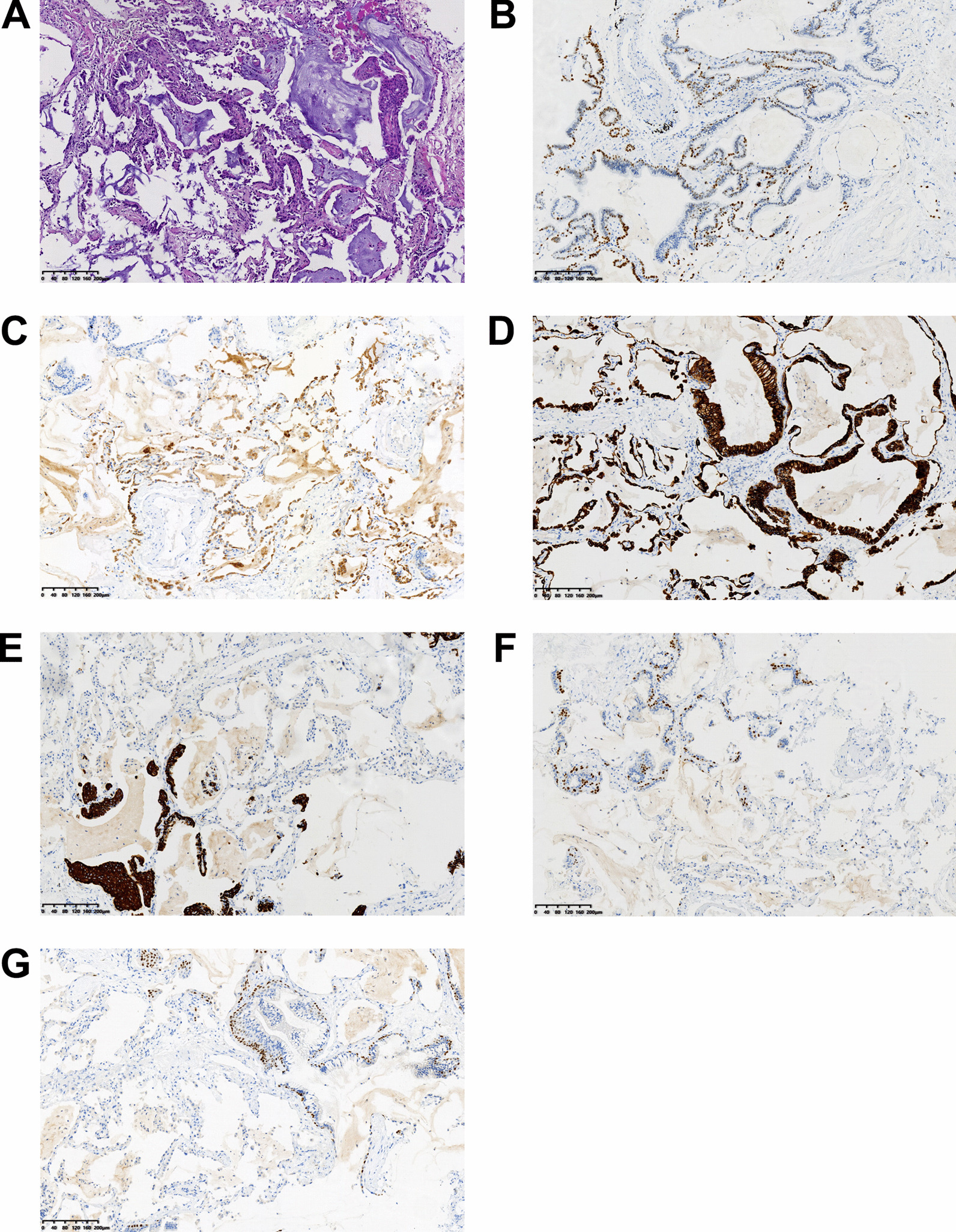
Representative H&E staining (**a**), IHC for TTF-1 (**b**), Napsin-A (**c**), CK7 (**d**), CK5/6 (**e**), P63 (**f**), and P40 (**g**) for biopsy samples. Original magnification. 100X

Initial ultrasound of the spleen and kidneys was unremarkable, although he did have chronic signs of hepatic steatosis detectable in the liver. The patient’s chest CT showed bilateral lung interstitial inflammation with edema (Fig. 1) but did not specifically reveal the presence of any detectable solid mass. Pulmonary function tests revealed restrictive disease. The lateral basal segment of the patient’s right lower lung lobe was biopsied via bronchoscopy. Histological analysis revealed abundant basal cells, ciliated columnar epithelial cells, and mucous cells in a disorganized pattern, which is characteristic of CMPT. Immunohistology indicated that the tissue was positive for TTF-1, Napsin-A, CK7, CK5/6, P63, and P40 (Fig. 2, Additional file 1: Fig. 1). Pathological analysis additionally indicates many lesions having features of adenomatous malignant transformation without breakthrough of the basement membrane. These findings lead to the diagnosis of ciliated muconodular papillary tumor accompanied with adenocarcinoma in situ.

The patient was encouraged to eat a low-sodium and low-fat diet due to his chronic hepatic steatosis and prescribed a statin medication for his hyperlipidemia. The patient was put on a ventilator until his oxygen saturation on room air reached 95%. This patient did not have a solid resectable lesion identifiable on CT scan; therefore, surgery was not performed. For the adenocarcinoma in situ, adjuvant chemotherapy was performed, as the lesion was disseminated in both lungs. The patient was treated with the chemotherapeutic drugs cisplatin and pemetrexed [8]. A strategy of wait and watch was applied for CMPT. The patient’s symptoms improved, and he was discharged with close follow up.

## Discussion and conclusions

CMPT is a very rare disease; CMPT accompanied by adenocarcinoma in situ has not previously been reported. In this study, we reported a unique case of CMPT accompanied with a malignant tumor, adenocarcinoma in situ. The specific cause of our patient’s tumor is not clear, which is consistent with previous case reports [2, 7, 9].

Many of the cases of CMPT reported in literature have appeared in patients residing in eastern Asia [2, 4, 5], and if a genetic background plays a role here, this link is not clear. This prevalence possibly could be due to the association of TTF-1 (thyroid transcription factor 1) mutations with EGFR (epidermal growth factor receptor) mutations in lung tissue [5]. This association was seen in East Asian populations with an odds ratio (OR) of 4.33, 95% CI: 3.46–5.41, p < 0.00001 [5]. Nearly half of all patient’s diagnosed have a history of tobacco smoking^1^. The tumor appears to affect both men and women equally [2]. The median age of onset for CMPT is 67 years old [2], however a case of CMPT in a 19-year-old patient has been documented in the past [9]. Our patient is a 53-year-old, resides in China, and has an extensive history of smoking.

Histological analysis of the tumor is necessary and critical for definitive diagnosis of CMPT. Based off the retrospective 38 case analysis by Dr. Lu and Dr. Yeh, diagnosis of CMPT is determined from presence of basal cells, ciliated columnar epithelial cells, and mucous cells in a disorganized glandular, papillary, or micropapillary pattern [2]. Immunohistochemical analysis of our patient’s tumor was positive for TTF-1 and CK7, both of which are common findings for CMPT [2, 9]. Our patient also had markedly elevated carcinoembryonic antigen, a common tumor pathological finding that has been identified in previous CMPT cases [2]. The presence of basal cells, determined via P40 and CK5/6 staining, is a definitive characteristic of CMPT [10]. Staining for presence of basal cells can be used to distinguish CMPT from pulmonary adenocarcinoma [11]. Napsin-A analysis was positive for our patient’s tumor as well, which does not appear to have been a component of previous case reports. Lastly, the presence of relatively large amounts of mucin near the tumor are positive indicators for CMPT diagnosis [2, 9].

CT imaging in previous cases typically does not lend much to the eventual specific diagnosis of CMPT, rather than only revealing the presence of an actual tumor. CT findings in previous cases typically show small, peripherally located lesions, and these lesions can vary in morphology [2]. In other reported cases, these tumors were most often found as an incidental finding on CT [12]. Our patient’s CT did not specifically reveal the presence of a definitive, solid lesion, but rather a pattern of interstitial inflammation and edema with diffuse, patchy, ground glass opacities in both lungs. Some of these lesions presented with a network structure. Compared with imaging findings in previous reports of CMPT, this patient’s CT appears to be similar, as other publications document the presence of ground glass opacities [2, 12]. CMPT was eventually diagnosed on tracheoscopy with immunohistological analysis.

If there is a tumor present, the most common treatment of CMPT is via surgical excision. The most common surgical technique is wedge resection, although multiple lobectomy procedures have been used in the past to treat CMPT as well [2]. In the past, due to a lower level of understanding of CMPT morphology, a greater number of procedures were performed because more severe disease was suspected. Regardless of whether partial resection, wedge resection, or lobectomy was performed, there have been no signs of surgical complication or adverse outcomes [2]. Fortunately, prognosis following removal of CMPT has also been very positive, with no documented history of recurrence or metastatic disease after treatment [2, 4, 11, 12]. For this reason, we believe minimally invasive procedures with or without adjuvant therapy are warranted for treatment of CMPT that is not accompanied by adenocarcinoma in situ.

Much of the pathophysiology of CMPT has yet to be uncovered according to multiple current literature studies [2, 3, 6]. In this case, our patient’s diagnosis with CMPT was accompanied with adenocarcinoma in situ. Given the current knowledge available regarding CMPT, this is a unique circumstance in which CMPT has been linked to malignancy. Further research needs to be performed to investigate the potential relationship between CMPT and a potential for malignancy transformation.

With the advance in imaging and diagnostic techniques, coupled with the increasing body of knowledge and awareness for this rare disease, it is expected that more patients will be correctly diagnosed with CMPT in the future. In addition, the potential for CMPT to be linked with a malignancy appears to be a new development that requires careful consideration when caring for other patients who receive a diagnosis of CMPT.

## Supplementary Information

**Additional file 1**. Representative H&E staining for Fig. 2.

## Data Availability

The data are available from the authors upon reasonable request.

## Abbreviations

CMPT: Ciliated muconodular papillary tumor
CT: Computed tomography
WHO: World Health Organization
ABG: Arterial blood gas
CBC: Complete blood count
BMP: Basic metabolic panel
HIV: Human immunodeficiency virus
TTF: Thyroid transcription factor
CK: Cytokeratin
EDGF: Epidermal growth factor receptor
OR: Odds ratio
CI: Confidence interval
