# Supplementary figures and images for: A rare case of ciliated muconodular papillary tumor accompanied with adenocarcinoma in situ
Source: BMC Pulm Med. 2021 Jul 12;21:223. doi: 10.1186/s12890-021-01581-9 (PMC8273990; doi:10.1186/s12890-021-01581-9)

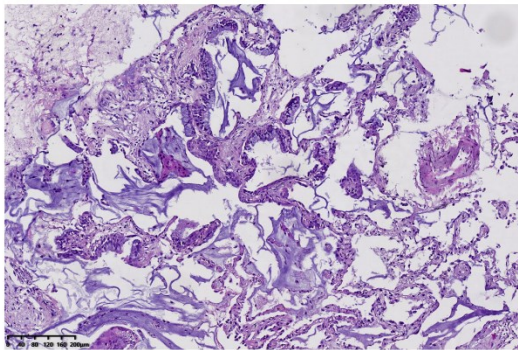

**Supplemental Figure:** Representative H&E staining for Fig 2.

Supplement: Supplementary file 1 — Additional file 1. Representative H&E staining for Fig. 2. [file 12890_2021_1581_MOESM1_ESM.pdf]
